# Mushrooms-Rich Preparations on Wound Healing: From Nutritional to Medicinal Attributes

**DOI:** 10.3389/fphar.2020.567518

**Published:** 2020-09-16

**Authors:** Javad Sharifi-Rad, Monica Butnariu, Shahira M. Ezzat, Charles Oluwaseun Adetunji, Muhammad Imran, Seyyed Reza Sobhani, Tabussam Tufail, Tahereh Hosseinabadi, Karina Ramírez-Alarcón, Miquel Martorell, Alfred Maroyi, Natália Martins

**Affiliations:** ^1^ Zabol Medicinal Plants Research Center, Zabol University of Medical Sciences, Zabol, Iran; ^2^ Banat’s University of Agricultural Sciences and Veterinary Medicine “King Michael I of Romania”, Timisoara, Romania; ^3^ Pharmacognosy Department, Faculty of Pharmacy, Cairo University, Cairo, Egypt; ^4^ Pharmacognosy Department, Faculty of Pharmacy, October University for Modern Sciences and Arts (MSA), 6th of October, Dokki, Egypt; ^5^ Applied Microbiology, Biotechnology and Nanotechnology Laboratory, Department of Microbiology, Edo University Iyamho, Auchi, Nigeria; ^6^ Faculty of Allied Health Sciences, University Institute of Diet and Nutritional Sciences, The University of Lahore, Lahore, Pakistan; ^7^ Department of Community Nutrition, Faculty of Nutrition Sciences and Food Technology, Shahid Beheshti University of Medical Sciences, Tehran, Iran; ^8^ Department of Pharmacognosy and Biotechnology, School of Pharmacy, Shahid Beheshti University of Medical Sciences, Tehran, Iran; ^9^ Department of Nutrition and Dietetics, Faculty of Pharmacy, University of Concepción, Concepción, Chile; ^10^ Centre for Healthy Living, University of Concepción, Concepción, Chile; ^11^ Unidad de Desarrollo Tecnológico, UDT, Universidad de Concepción, Concepción, Chile; ^12^ Department of Botany, University of Fort Hare, Alice, South Africa; ^13^ Faculty of Medicine, University of Porto, Porto, Portugal; ^14^ Institute for Research and Innovation in Health (i3S), University of Porto, Porto, Portugal

**Keywords:** mushrooms, bioactive compounds, ethnobiology, ethnopharmacology, wound healing

## Abstract

Mushrooms have a significant role in human diet as functional food and as a nutraceutical resource. The combination of its umami flavor, protein, vitamins, minerals and carbohydrates has meant that mushrooms could be considered a cheap food source for a long time in many countries. Moreover, mushrooms contain an excellent variety of bioactive metabolites that can be successful in both prevention and treatment of various human health hazards. In addition, extracts from medicinal mushrooms and their metabolites have been verified for wound treating with contribution to different mechanisms of the healing process. This review summarizes the nutritional value and composition of mushrooms, ethnobiology and ethnopharmacology, and wound healing potential.

## Introduction

Mushrooms are macrofungi, including mostly basidiomycetes and some ascomycetes species (Chang and Miles, 2004; Lindequist et al., 2005; Wang et al., 2014). The estimated number of mushrooms species in the earth is 140,000 (Kim, 2017), comprising around 22,000 identified species (Lindequist et al., 2005). From long times ago mushrooms were extensively used worldwide for their nutritional and medicinal features, predominantly in some Asian, Northern and Central American countries (Badalyan, 2014).

The cultivation of fruiting body and mycelium, the production of bioactive components in short time and the possibility of manipulation to optimize production are some of the most renowned mushrooms’ advantages as a source of functional foods and beneficial natural molecules (Ferreira et al., 2009). From the perspective of nutrient source, mushrooms contain high protein and low-fat contents, along with a high amount of vitamins B, C, D, and K, minerals, including potassium and phosphorus, and also selenium as a trace element. They have also considerable amounts of dietary fibers (Badalyan, 2014), chitin and β-glucans as functional components (Manzi et al., 2001).

The main medicinal properties ascribed to mushrooms include antibiotic, antitumor, antiviral, immunostimulant and hypolipidemic activities (Chowdhury et al., 2015). Several researchers have reported anti-elastase, anti-collagenase, anti-hyaluronidase and anti-tyrosinase activities in mushroom extracts and their isolated compounds (Taofiq et al., 2016; Zengin et al., 2017). In the same line, wound healing promotion has been one of the most studied beneficial effects of mushrooms, described in different scientific literatures (Schepetkin and Quinn, 2006; Cheng et al., 2013). Mushrooms’ medicinal properties are closely linked to its content in bioactive compounds, which mostly included polysaccharides, terpenoids, glucans, phenolic compounds, statins, lectins, among others. Melanin pigments, chitin and chitosan which are found in their cell wall along with extracellular enzymes are other effective molecules (Lindequist et al., 2005; Badalyan, 2014; Ruthes et al., 2016). The wound healing properties of mmushrooms include different mechanisms such as immune epithelial cells stimulation, and cytokines and growth factors release (Amin et al., 2015; Krupodorova et al., 2015). Thus, given the above listed aspects, the present review aimed to provide key insights on mushrooms nutritional value and composition, ethnobiology and ethnopharmacology, and wound healing potential.

## Mushrooms: Nutritional Value and its Role in our Diet

### Mushrooms: The Role in Our Diet

A balanced and healthy diet includes plants, plant-derived products, and mushrooms (Reis et al., 2017). Their production and consumption have continuously increased over time due its nutritional value and health benefits (Aida et al., 2009; Reis et al., 2017). Nowadays, the use of mushrooms has markedly grown in pharmaceutical, nutraceutical, and cosmeceutical industries (Rathore et al., 2017). Indeed, edible mushrooms have a high nutritional value, more than any vegetable or fruit (Manjunathan and Kaviyarasan, 2011), and have been eaten for their flavor, economic and ecological benefits, and therapeutic properties for many years (Sánchez, 2017). Their texture and enjoyable flavor properties make them a feasible alternative for meat. Its low energetic density, with about 92% water, allows to reduce the energetic density of the final meal, when mushrooms are used instead of other higher energy-dense ingredients (Feeney et al., 2014b). The most cultivated and consumed mushrooms worldwide is *Agaricus bisporus*, followed by *Pleurotus* spp. and *Lentinula edodes* (Aida et al., 2009; Roncero-Ramos and Delgado-Andrade, 2017).

### Energetic Value and Macronutrients Content

Due to their low-fat content, mushrooms are low in calories (Muszyńska et al., 2018). The total energetic value of mushrooms is between 250 and 350 calories/kg of fresh mushrooms (Panneerselvam et al., 2009; Sánchez, 2010). The dry matter of mushrooms comprise around 50% to 65% total carbohydrates, 19% to 35% proteins, and 2% to 6% fat (Rathore et al., 2017). Edible mushroom comprises a maximum number of all essential amino acids (Hayes, 1976), with arginine, glutamic acid, and aspartic acid being the most abundant ones (Manzi et al., 1999). Thus, mushrooms can be considered an great alternative to animal products (Dembitsky et al., 2010). Indeed, tryptophan and lysine, which are absent in vegetables, are present at high concentrations when compared to cysteine and methionine (Murugesan, 2017).

Alcoholic sugars, like mannitol and trehalose, are some carbohydrates components present in mushrooms (Jain and Roy, 2009). Regarding fats, mushrooms are rich in polyunsaturated, monoinsaturated and saturated fatty acids, as palmitic, oleic, and linoleic acids (Kavishree et al., 2008; Muszyńska et al., 2018).

### Vitamins and Minerals

Mushrooms are rich in niacin (B3), pantothenic acid (B5) and riboflavin (B2) (Feeney et al., 2014b; Murugesan, 2017), but other vitamins, such as thiamine (B1), pyridoxin (B6), cobalamin (B12), biotin (B7) and D have also been reported (Pankavec et al., 2019; Rózsa et al., 2019). The only non-animal-based food that contains vitamin D are mushrooms (Feeney et al., 2014b; Rathore et al., 2017), and curiously the content of vitamin B12 in mushrooms is similar to that reported for beef, liver, and fish (Feeney et al., 2014b).

Moreover, mushrooms are also rich in minerals, such as potassium, calcium, phosphorus, and magnesium. Because of its low sodium and high potassium concentration, they have a Na^+^/K^+^ ratio less than 0.6, being thus a good food option amongst other vegetables for hypertense individuals (Nieman et al., 1992; Rajarathnam et al., 1998). Indeed, mushrooms are an excellent source of selenium and copper, with high bioavailability (Feeney et al., 2014b; Murugesan, 2017). Mushrooms have also an interesting content in tocopherols, carotenoids, ascorbic acid, phenolics, and ergosterol, being even most abundant than that found in most vegetables and fruits (Sánchez, 2017).

On the other side, mushrooms are also a good source of different dietary fiber, including β-glucans, polysaccharide-protein complexes (PSPC), chitin, hemicelluloses, mannans, and xylans (Pardeshi and Pardeshi, 2009; Rathore et al., 2017). Thus, edible mushrooms consumption can easily provide up to 25% of the recommended dietary fiber intake (Cheung, 2010).

### Secondary Metabolites in Mushrooms

Several bioactive compounds are present in mushrooms, such as cinnamic acid, ergosterol, and *p*-coumaric, *p*-hydroxybenzoic and protocatechuic acids. The bioactive compounds with special pharmacological value present in shiitake mushroom are lentinan [C_42_H_72_O_36_], eritadenine or lentinacin [C_9_H_11_N_5_O_4_], and ergotamine [C_33_H_35_N_5_O_5_] (Ribeiro et al., 2006).

### A Sustainable Food Choice

As a result of its ability to control environmental factors, land use, and related energy and water needs during growing mushrooms, its production is consistent with sustainable food supply (Feeney et al., 2014b). Indeed, mushrooms have a small environmental footprint and require relatively little water content or land (Feeney et al., 2014a). Therefore, mushroom can be considered a sustainable food choice.

## Mushrooms: From Ethnobiology to Ethnopharmacology

Mushrooms contain a fruiting body collected of a pileus (cap), lamellae (gills), stipe (stalk), and mycelium, the root absorb the nutrients (Verma et al., 2019). In historic times, mushrooms were considered part of the plant kingdom, but the present taxonomy recognizes fungi as an independent group of organisms under the *Mycota* kingdom, generally due the presence of chitin inside their cell walls. Although in wild mushrooms are seasonal and can be collected and used, they can be domesticated through spore culture.

Mushrooms are mysterious in their composition, containing umami-flavored, some medicinal, some hallucinogenic/psychoactive, while some poisonous compounds. Additionally, various fungal species also accumulate heavy metals in their fruit bodies. Currently, the most commonly used mushrooms as significant health boosters include Chaga (*Inonotus obliquus*), Cordyceps (*Cordyceps sinensis*), Lion’s Mane (*Hericium* *erinaceus*), Reishi (*Ganoderma lucidum*), Shiitake (*L. edodes*), and Turkey Tail (*Trametes versicolor*).

Mushrooms are an inexhaustible source of immunomodulating biomolecules, whose effects in the treatment of cancer, infectious or immunological disorders have already been clinically demonstrated (Yang et al., 2013; Dai et al., 2015). Mushrooms contain immunostimulant compounds, such as β-glucans, polyinosinic: polycytidylic acid and lip polysaccharide, that makes stronger and boost the immune system activity. Moreover, mushrooms folate plays an important role in DNA synthesis and repair (Trovato et al., 2017). At present, there are over 270 recognized fungal species for their various biological activities (such as adaptogenic, anti-inflammatory, antimicrobial, antioxidant, hepatoprotective, hypocholesterolemic). As their cultivation has grown for food purposes, mushrooms have become increasingly used as dietary supplements against various diseases and associated health problems (Ferreira et al., 2009). Additionally, their anti-atherogenic, hepatoprotective, antinociceptive, anti-inflammatory, antidiabetic and antioxidant effects have also been reported (Lindequist et al., 2005; Badalyan, 2014; Ruthes et al., 2016). In last decades, several scientific researches performed in Japan, China, and Korea, and lately in the United States, have proved the mushrooms potential for the prevention and treatment of some chronic diseases, like cancer and hypertension (Manzi et al., 2001; Kimura, 2013) and heart disease (Brzezicha-Cirocka et al., 2019). At the present, in some Asian countries several mushroom extracts are consumed in cancer treatment as adjuvant care in addition to other therapies (Chowdhury et al., 2015).

Internationally, the consumption of various mushrooms species in the form of dietary supplements has been approved by a number of specialized organizations, such as European Novel Food Regulations, Complementary Medicine in the UK, National Health Service, German Federal Health Agency; Japan - Foods for Specific Health Use (FOSHU) - the largest in the world; USA - Federal Food Drug and Cosmetic Act, Dietary Supplement Health and Education Act (DSHEA), National Institute of Health (NIH), and American Herbal Products Association (AHPA) (Roupas et al., 2012). Most studies have been conducted by specialized centers in the treatment of various cancers, acquired immunodeficiency syndrome (AIDS) and other illnesses caused by an imbalanced immune system. Studies on some mushroom species have also shown their ability to support the body in the interferon’ synthesis (Matuszewska et al., 2019).

## Mushrooms Effects: Emphasis on Wound Healing Potential

**Figure 1:**
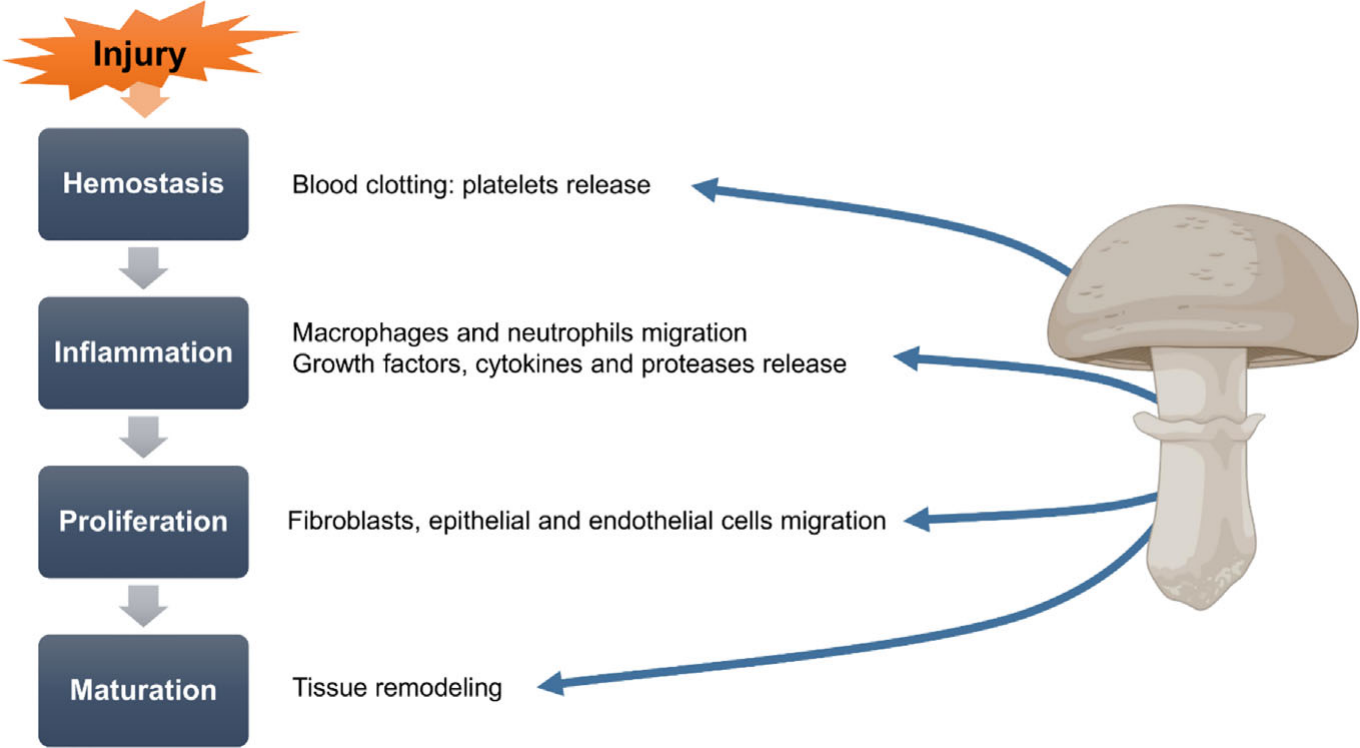
Role of mushrooms in the wound healing process.

Wound healing as a normal biological process, that may be divided into 4 phases: blood clotting (hemostasis), inflammation, tissue growth (proliferation), and tissue remodeling (maturation) (Guo and Dipietro, 2010; Amin et al., 2015). Wound repair involves many cell types populations and molecular mediators, and is consented as a physiological mechanism using the immune system, the complex skin repair process (Abdulla et al., 2010; Chen et al., 2020). Several factors can repair ulcers, affecting one or more phases in the process (Guo and Dipietro, 2010). Briefly, wound healing involves an increase in collagen deposition (Portera et al., 1997) and regulation of balance and occlusion by blood platelets (Li et al., 2012). Medicinal mushroom extracts and their metabolites have been verified for wound treating, with contribution in different mechanisms, including immune epithelial cells stimulation, extracellular matrix, cytokines, growth factors, reactive oxygen species (ROS), and various inﬂammatory intermediates (Amin et al., 2015; Krupodorova et al., 2015). The efficiency of wound healing process is largely dependent of the balance of proinflammatory and proregenerative signals, which are mediated by cytokines (Sureda et al., 2016; Nosenko et al., 2019). **Figure 1** summarizes the wound healing process and the role of mushrooms in the different steps.

Browder et al. (1990) extensively studied glucan as a potent macrophage stimulant. The 1→3 β-glucans of mushrooms may recruit macrophages to the wound site as mechanism of wound healing. In fact, β-glucans is thought to mediate effects through activation of macrophages, neutrophils, natural killer (NK) cells, and lymphocytes. Moreover, it has been reported an immunostimulatory activity and enhance wound healing of β-glucans by increasing macrophage infiltration into the injury sites and stimulating tissue regeneration (Minutti et al., 2017). In addition, β-glucans directly increase the synthesis of types I and III collagen (Kwon et al., 2009; Cheng et al., 2013), stimulating the collagen regeneration and helping the wound healing (Portera et al., 1997). The use of β-glucan in combination with collagen matrix wound dressing reduced the requirement of various analgesics and dressing changes in partial-thickness burns in children (Delatte et al., 2001). In a rat model, the combination of β-glucans from mushroom with poly vinyl alcohol speeded up the wound healing time by 48% (compared to cotton gauze) (Huang and Yang, 2008). In induced ulceration rats, β-glucan obtained from both *Ganoderma lucidum* and *Lentinus edodes* raised serum antioxidant enzymes activities and decreased levels of serum mucosal interleukin-2 (IL-2) and tumor necrosis factor (TNF)-α (Gao et al., 2004a; Gao et al., 2004b; Yu et al., 2009). The suppression of TNF-α enhances wound healing increasing epithelial cell proliferation and gastric blood flow and decreased epithelial apoptosis (Gao et al., 2002). Water-soluble β-glucan obtained from *Piptoporus betulinus* fruiting bodies promoted cell migration on *in vitro* scratch assay on Caco-2 cells (De Jesus et al., 2018). Epithelial cell migration seems to be orchestrated by integrin-induced FAK/Src signaling pathway (Zhao et al., 2016). β-Glucans from *Schizophyllum commune* provoked re-epithelialization through integrin/FAK/Src signaling pathway and activated dermal transformation *via* integrin or ROS production (Seo et al., 2019).

In short, the wound healing property of mushrooms in many studies have been moderately associated to their rich content in polysaccharides. For example, *G. lucidum* aqueous extract, *Ganoderma tsugae* (Chien et al., 2008) and polysaccharides form *Agaricus blazei* and *Phellinus gilvus* (Bae et al., 2005b) have been reported as useful agents in wound healing with several associated mechanisms (Krupodorova et al., 2015). The polysaccharides from *G. lucidum* can bind through specific receptors or serum-specific proteins to leucocyte surfaces and alter their activities of macrophage, T-helper, NK cells, and other effector cells (Müller et al., 1996; Mueller et al., 2000). Moreover, *G. lucidum* polysaccharides are potent immune-modulating agents (Gao and Zhou, 2002). The polysaccharides of *P. gilvus* decreased IL-1 expression in skin of burn wound-treated rats (50 or 100 mg/kg b. w.) (Sui et al., 2010). *P. gilvus*-isolated polysaccharides reduced wound contraction and enhanced the re-epithelialization of the 6-mm circular wounds in streptozotocin (STZ)-induced diabetic rat by topical application twice a day for 5 days (Bae et al., 2005a). *G. lucidum* hot aqueous extract was standardized to contain total polysaccharides (25.1%), ganoderic acid A (0.45%), and adenosine (0.069%) (Cheng et al., 2013). A 10% aqueous extract cream of this mushroom was tested on wounds in the posterior neck region of STZ-induced diabetic rats. The mushroom extract accelerated wound closure more than the standard Intrasite gel. Moreover, the antioxidant activity was significantly higher in the serum of treated rats, and oxidative markers, proteins and lipids were lowered. Another study by Gupta et al. (2014) has shown that the aqueous lyophilized extract of *G. lucidum* from the Indian Himalayan region promoted the healing efficacy on wound dermal excision in experimental rats. Ling Zhi-8 (LZ-8) is an immunomodulatory protein isolated from *G. lucidum* mycelia (Lin et al., 2014). Ten µL of 1 mg/ml LZ-8 protein solution dropped on the wound in rat liver tissues after monopolar electrosurgery significantly increases wound healing and decreased nuclear factor-κB (NF-κB), caspase-3 and apoptosis. The *in vivo* wound healing activity of aqueous extracts of *G. lucidum* and *Crinipellis schevczenkovi* (100 mg powder mycelium/1 ml water for injections) were investigated using the excision wound healing model on albino mice line (Krupodorova et al., 2015). The topical application of *C. schevczenkovi* mycelium extract induced wound healing on 3^th^ day, while on the 6^th^ day, both extracts induced a complete healing (8^th^ day in control group).

*H. erinaceus*, with a rich content on β-glucan polysaccharides, have exhibited wound healing acceleration in experimental rats (Khan et al., 2013). *H. erinaceus* freeze-dried fruiting bodies reduced ulceration as pre-treatment (250 and 500 mg/kg b.w.) in ethanol-induced gastric ulcers in rats (Abdulla et al., 2008). In addition, the oral administration of the medicinal mushroom *Sparassis crispa* (>40% β-glucans) at a dose of 1.000 mg/kg b.w. per day, for 4 weeks, significantly accelerated the wound closure through increasing the collagen regeneration, macrophage and fibroblast migration, and wounds epithelialization in STZ-induced diabetic rats (Kwon et al., 2009). The receptors of β-glucans are present in human dermal fibroblasts making these can receive direct signals from glucans promoting wound healing not only by activating macrophages, but also by stimulating fibroblasts (Kougias et al., 2001). After β-glucan stimulation, fibroblasts activate two translation factors, activator protein-1 (AP-1) and specific protein-1 (SP-1), and two signaling pathways, NF-κB and nuclear factor-1 (NF-1), to enhance the immune response, hyperplasia and the expression of collagen precursor genes (Kougias et al., 2001; Lowe et al., 2002).

*Antrodia camphorata* extract, rich in total polyphenols and flavonoids, have exhibited noteworthy wound healing effects in rats, leading to an increase in wound constriction and collagen accumulation. In another study, *A. camphorata* extract significantly promoted wound healing *in vivo* by observation of less inﬂammatory cells and more collagen synthesis in the injured tissues, and also *in vitro* by promoting fibroblast cells proliferation (Amin et al., 2015). Furthermore, *A. bisporus*, rich in polysaccharides, phenolic compounds and lectin, has also been reported effective in controlling wound procedures afterward glaucoma surgery (Krupodorova et al., 2015). The edible mushroom lectin from *A. bisporus* tested on Tenon’s capsule fibroblasts *in vitro* models of wound healing showed a dose-dependent inhibition of proliferation and lattice contraction (Batterbury et al., 2002). More studies related with wound healing activities of mushrooms are shown in **Table 1**.

**Table 1:** Wound healing activities of mushrooms.

Mushroom spp.	Family	Bioactive components	Properties/Mechanism of action	References
*Agaricus bisporus*	*Agaricales*	Lectin (0–100 mg/ml)	Dose-dependent inhibition of proliferation and lattice contraction in an *in vitro* model	(Batterbury et al., 2002)
*Agaricus blazei*	*Agaricales*	Polysaccharides	Impact on interleukin (IL)-1 expression in skin of burn wound-treated rats (50 and 100 mg/kg b. w.)	(Sui et al., 2010)
*Agaricus sylvaticus*	*Agaricales*	Crude extract (proteins, phenols, β-glucans, phenols, chlorogenic, caffeic, coumaric and benzoic acids)	Enhanced wound healing in Wistar rats (1 ml of gel at 10% *Agaricus sylvaticus* for 14 days). Phenols promoted the healing process	(Da Silva et al., 2018)
*Auricularia auricula*	Auriculariaceae	Polysaccharides extracts (0.1, 1 and 10 mg/ml)	Wound healing effect with 100 μg per wound for *A. auricula* (140.43%) in *ex vivo* porcine skin wound healing model	(Khamlue et al., 2012)
		Acidic polysaccharides	Anticoagulant effects by improving catalysis of thrombin reticence by antithrombin and prevention of platelet aggregation	(Yoon et al., 2003)
*Calvatia gigantean*	Agaricaceae	Unspecified	Mushrooms consumption enhanced wound healing. Used for curing stomach upset and to cure stomach pains in woman during menstruation.	(Thangaraj et al., 2017)
*Crinipellis schevczenkovi*	Agaricales	Powdered mushrooms mycelium (100 mg)	Promoted wound healing and histology of healed wounds on the 5^th^ day	(Krupodorova et al., 2015)
*Daldinia concentrica*	Hypoxylaceae	Crude extract (alkaloids, flavonoids, phenols, tannins, terpenoids, and saponins)	Increased wound healing activity in albino Wistar rats by excision wound model	(Thangaraj et al., 2017)
*Fomes fomentarius*	Polyporaceae	Unspecified	Ash combined with oil on skin ailments exerted wound healing promotion	(Malik et al., 2017)
*Ganoderma lucidum*	Basidiomycetes	Polysaccharides-rich extract	Topical application with 10% w/w enhanced wound healing in STZ-induced diabetic rats	(Cheng et al., 2013)
		Crude extract containing 5% v/v ethanol, absolute methanol and deionized water	Wound healing promotion. Primary and secondary triterpenes (0.156 mg/ml) from the methanol extract enhanced the healthy proliferation and migration of keratinocytes with no cytotoxic or aberrant morphological effects	(Montalbano, 2018)
		Polysaccharide fractions (0.1, 0.5, or 1.0 g/kg daily; intragastrically, for 14 days)	Marked healing promotion on acetic acid-induced ulcers in rats	(Gao et al., 2004b)
		Unspecified	Crude extract improved wound healing	(Dora and Hena, 2016)
		Unspecified	Crude extract decreased gastric ulceration in rats	(Rony et al., 2011)
		Immunomodulatory protein (Ling Zhi-8)	Accelerated wound healing in rat liver tissues after monopolar electrosurgery	(Lin et al., 2014)
		Powdered mushrooms mycelium (100 mg)	Increased wound healing activities and the histology of healed wounds on the 3^rd^ day	(Krupodorova et al., 2015)
		Aqueous lyophilized extract	In experimental rats improved wound healing, enhanced healing activity, evidenced by an increase in wound contraction, collagen accumulation, hexosamine, and total protein contents	(Gupta et al., 2014)
*Ganoderma praelongum*	Basidiomycetes	Unspecified	Crude extract enhanced wound healing	(Ameri et al., 2013)
*Hericium erinaceus*	Aphyllophoromycetidea	β-glucan polysaccharides	Accelerated wound healing in rats	(Khan et al., 2013)
		Unspecified	Crude extract (20, 30 and 40 mg/ml) accelerated wound healing enclosure in experimentally wounded male rats	(Abdulla et al., 2011)
		Unspecified	Crude extract enhanced wound healing	(Sokół et al., 2016)
*Lentinus edodes*	Marasmiaceae	Polysaccharide	Increased serum antioxidant enzymes activities and decreased serum mucosal IL-2 and tumor necrosis factor (TNF)-α levels in rats with oral ulceration. The protective action involve an oxidant mechanisms.	(Yu et al., 2009)
*Lycoperdon echinatum*	Agaricaceae	Unspecified	Crude extract enhanced wound healing	(Chang and Miles, 2004)
*Lycoperdon pusilum*	Agaricaceae	Unspecified	Crude extract enhanced wound healing	(Rai et al., 2005)
*Phallus impudicus*	Phallaceae	Unspecified	Mushroom consumption enhanced wound healing	(Malik et al., 2017)
*Phellinus gilvus*	Hymenochaetaceae	Polysaccharide	Increased dermal wound healing in STZ-induced diabetic rats by topical application twice a day for 5 days	(Bae et al., 2005a)
*Pisolithus arhizus* (Scop.) Rauschert	Sclerodermataceae	Unspecified	Crude extract enhanced wound healing	(Mohamed and Dix, 1988)
*Pleurotus sapidus*	Pleurotaceae	Unspecified	Crude extract enhanced wound healing	(Thimal and Kluthe, 1998)
*Podaxis pistillaris*	Agaricaceae	Unspecified	Mushrooms consumption enhanced wound healing	(Thangaraj et al., 2017)
*Sparassis crispa*	Sparassidaceae	Unspecified	Oral administration improved wound healing in mice with STZ-induced diabetes	(Yamamoto and Kimura, 2013)
		β-glucan component	Wound healing promotion in STZ-induced diabetic rats	(Kwon et al., 2009)
		Oral administration of *Sparassis crispa* (1,000 mg/kg b.w./day for 4 weeks)	Accelerated wound healing in rats with STZ-induced diabetes and effectively promoted wound healing in diabetic patients	(Kwon et al., 2009)
*Termitomyces heimii*	Lyophyllaceae	Unspecified	Crude extract enhanced wound healing	(Chandrawati and Narendra Kumar, 2014)
*Termitomyces microcarpus*	Lyophyllaceae	Fresh extract powder/paste of fruiting bodies	Increases wound healing	(Aryal and Budhathoki, 2015)
		Unspecified	Crude extract enhanced wound healing	(Chandrawati and Narendra Kumar, 2014)
		Unspecified	Mushrooms consumption enhanced wound healing	(Thangaraj et al., 2017)
*Tremella fuciformis*	Tremellaceae	Polysaccharides extracts (0.1, 1 and 10 mg/ml)	Wound healing promotion Wound healing effect with 1 μg per wound for *T. fuciformis* (125.38%) in *ex vivo* porcine skin wound healing model	(Khamlue et al., 2012)
*Russula delica*	Russulaceae	Unspecified	Crude extract enhanced wound healing	(Turkoglu et al., 2007)
*Scleroderma citrinum*	Sclerodermataceae	Unspecified	Crude extract enhanced wound healing	(Wakefield, 1964)

## Concluding Remarks and Upcoming Perspectives

Mushrooms have been eaten for many years due to their flavor, economic and ecological benefits, and therapeutic properties. With the research advances, numerous bioactive compounds obtained from mushrooms have been looked as responsible for their biological and therapeutic properties, with their anti-atherogenic, hepatoprotective, antinociceptive, anti-inflammatory, antidiabetic and antioxidant effects being the most remarkable ones. Nonetheless, the wound healing potential of mushrooms have also been increasingly reported, linked to their richness in polysaccharides and phenolic compounds. However, some species can cause extremely serious, sometimes deadly poisonings, being thus of extreme relevance to deepen knowledge on this field. Thus, further studies are needed to assess the mushrooms-derived bioactive compounds potential and their healing properties, as well as of the extracts from which they are derived. Nevertheless, the use of mushroom components and extracts in cosmetics may be considered a niche of attention, due to their remarkable wound healing properties.

## Author Contributions

All authors contributed equally to the manuscript. Conceptualization: JS-R. Validation investigation, resources, data curation, writing: all authors. Review and editing: JS-R, MM, AM, and NM. All authors contributed to the article and approved the submitted versión.

## Funding

This work was supported by CONICYT PIA/APOYO CCTE AFB170007.

## Conflict of Interest

The authors declare that the research was conducted in the absence of any commercial or financial relationships that could be construed as a potential conflict of interest.
